# Catastrophic musculoskeletal injuries in Thoroughbred racehorses on racetracks in Gauteng, South Africa

**DOI:** 10.4102/jsava.v90i0.1640

**Published:** 2019-02-28

**Authors:** Keith E. Spargo, Luis M. Rubio-Martinez, Dale P. Wheeler, Lizelle Fletcher, Ann Carstens

**Affiliations:** 1 Department of Companion Animal Clinical Studies, University of Pretoria, South Africa; 2 Department of Equine Orthopaedics and Surgery, University of Liverpool, United Kingdom; 3 National Horseracing Authority of Southern Africa, Turffontein, South Africa; 4 Department of Statistics, University of Pretoria, South Africa

## Abstract

The incidence and types of catastrophic musculoskeletal injuries in Thoroughbreds that resulted in euthanasia on selected racetracks in South Africa between 1998 and 2012 were investigated by an observational retrospective investigation. Data from the National Horseracing Authority of Southern Africa for these racetracks were used to calculate incidence rates in Thoroughbreds (*n* = 114) that sustained catastrophic musculoskeletal injuries during racing that required immediate euthanasia, based on the diagnosis made by the on-site veterinarian as well as on fetlock radiographs and dissections of 53 cadaver limbs of horses that sustained a catastrophic musculoskeletal injury. The proximal sesamoid bones and the distal suspensory ligament were involved in 55.26% of horses; 73.58% of the cadaver limb radiographs were of the left forelimb, 64.15% were closed fractures, and 74.47% had biaxial proximal sesamoid bone fractures. Catastrophic musculoskeletal injuries occurred almost exclusively unilaterally and involved mostly the left front leg. The average incidence rate for a catastrophic musculoskeletal injury occurring in a year at any of the tracks was 0.6 of 1000 starts.

## Introduction

Worldwide the racing industry is a multimillion-dollar business and injuries to horses are costly and detrimental to the welfare of the horse. There is a paucity of published information on the incidence and types of catastrophic musculoskeletal injuries (CMI) in Thoroughbreds on racetracks in South Africa. Catastrophic musculoskeletal injuries appear to be the most common type of injury ending a racehorse’s career and have been reported in most of the major racing countries including the UK, USA, Japan, Hong Kong, Australia and Canada (Arthur 2010; Bailey et al. 1998a, 1998b; Cruz et al. 2007; Hill 2003; Hitchens, Hill & Stover 2016; Lam, Parkin & Riggs 2007; Parkin et al. 2004; Wylie et al. 2017). However, only one study reported on the incidence rates of CMIs in South Africa (Macdonald et al. 2009).

The objectives of this study were to describe and report the types of CMIs that resulted in immediate euthanasia and to determine the incidence rate of CMIs in racing Thoroughbreds in South Africa

## Materials and methods

An observational retrospective investigation on horses that sustained CMIs resulting in immediate euthanasia during racing on selected racetracks from 1998 to 2012 was conducted by analysing data (total number of horses that raced during the study period on each racetrack, the signalment, racing data and the racetrack veterinarian’s report for all 114 horses that sustained a CMI during a race in the study period) obtained from the records of the National Horseracing Authority of Southern Africa (NHRA).

Catastrophic musculoskeletal injury was defined as a racing injury resulting in termination of the horse’s racing career because of a hopeless prognosis for future racing, as determined by an official racetrack veterinarian, and/or if the injury severity necessitated immediate euthanasia on the track or immediate vicinity. The racetracks (type of track in brackets) included in the study were Turffontein (turf), Gosforth Park (turf), Newmarket (turf) and Vaal (turf and sand). Racing took place in a clockwise direction at all four racetracks.

All races were attended by either an official veterinarian from the NHRA or a certified private veterinarian who submitted reports on all injuries or lameness that occurred during the race meeting. Diagnoses on site were based on palpation only, by necessity were broad and only related to the bone that they occurred in (Reardon et al. 2014). The racing stewards also compiled reports at the conclusion of a race meeting, noting any horses that failed to meet their prerace expectations or were lame, injured or euthanised. All these reports were data captured and stored digitally. During the study period the affected limb of every horse that had an injury to its fetlock that resulted in euthanasia was severed through the middle carpal–tarsal joint, bagged and tagged with all the horse’s information and history and then shipped to and stored at the Equine Research Centre, Faculty of Veterinary Science, University of Pretoria, Onderstepoort, South Africa (ERC). Once at ERC it was data-captured and an up-to-date list of all received limbs was maintained digitally. When the analysis was conducted, all reports and the database maintained of injuries were evaluated and checked to ensure that all limbs that had been submitted had indeed been received.

Anatomical location and identification of the CMI were obtained from the racetrack veterinarian reports. Catastrophic musculoskeletal injuries affecting the suspensory apparatus were referred to as a combined failure of proximal sesamoid bones and/or the suspensory ligament and/or distal sesamoidean ligaments, as information in the records precluded further classification. Only the limbs of 52 horses (53 limbs from 52 horses, as one horse injured both forelimbs) out of 114 horses that sustained a CMI were submitted for further evaluation. The criterion for submission was obvious pathology to their fetlocks. These limbs were further examined at the ERC by radiography and anatomical dissection. Standard radiographic views (DP, LM, LM flexed, DLPMO and DMPLO) were made of the cadaver limbs and centred on the metacarpo and/or metatarsophalangeal joint with the limb positioned horizontally directly on the cassette and held in position with positioning aids. Further radiographs were obtained if the injury extended beyond the radiographic field. The limbs were then dissected, and soft tissues – the digital flexor, extensor tendons and suspensory ligament – were evaluated for pathology present such as tears, haemorrhaging and/or any change to their normal structure.

Data were gathered and analysed in a descriptive manner. Collective incidence, pertaining to all four racetracks, for each racing year and the incidence per racetrack were calculated using SAS (SAS/STAT 2014). Incidence = (number of CMIs/number of starts) × 1000. The number of CMIs × 1000 normalises the data to allow for more direct comparison between racetracks (Turner et al. 2012).

### Ethical considerations

This study’s protocol and design were evaluated by the University of Pretoria’s Animal Ethics Committee and approved on 28 October 2013 (protocol no. V020/13). The limbs used in this study were from cadaver horses who had been humanely euthanised on or at the racetrack as a result of sustaining an injury to a limb that could not be surgically repaired or that would not heal sufficiently so as to allow them to return to racing. All the cadaver limbs and racing data were obtained from the National Horseracing Authority of Southern Africa (NHRA) as part of their regulatory activities, and evidence can be provided stating the NHRA’s approval of the study and the publication of the article.

## Results

A total of 114 horses sustained a CMI in the study period. Of those, 111 horses had CMIs involving the limbs (110 unilateral and one bilateral), two horses sustained pelvic fractures and in one case the injury location was not recorded. The incidence rate of CMIs at each racetrack is given in Table 1.

**TABLE 1:** Incidence of catastrophic musculoskeletal injuries per racing year and racetrack.

Variable	No. of CMIs	No. of starts	Incidence per 1000 starts
**Race year**			
1998	6	14 608	0.41
1999	6	15 162	0.40
2000	8	16 178	0.49
2001	10	15 315	0.65
2002	8	14 637	0.55
2003	7	13 625	0.51
2004	14	14 117	0.99
2005	6	14 362	0.42
2006	4	13 687	0.29
2007	6	12 773	0.47
2008	12	14 003	0.86
2009	7	14 683	0.48
2010	5	15 482	0.32
2011	15	15 333	0.98
Total	114	203 965	0.56
Average	8	14 569	0.6
**Racetrack**			
Gosforth Park	4	11 772	0.34
Newmarket	18	34 115	0.53
Turffontein	51	77 207	0.66
Vaal	41	80 871	0.51
Total	114	203 965	0.56
Average	29	50 991	0.51

No., number; CMIs, catastrophic musculoskeletal injuries.

**FIGURE 1:**
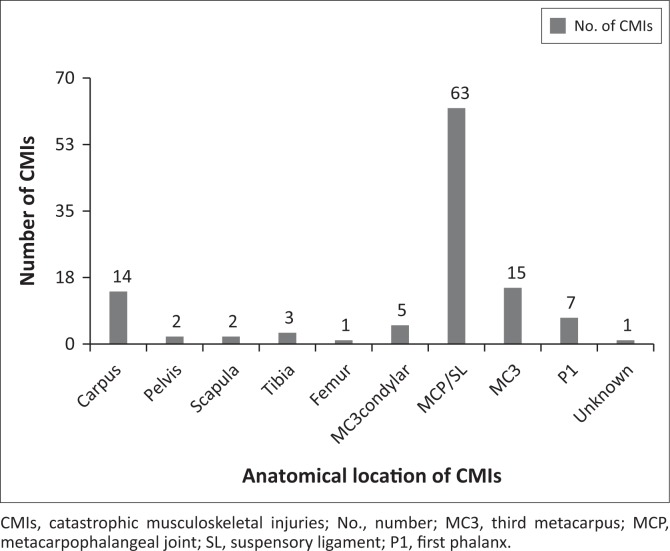
Bar chart depicting the anatomical location of the catastrophic musculoskeletal injuries incurred by 114 horses during the racing year period 1998–2012.

In 62 of the 114 horses (54.39%) the left forelimb (LF) was affected; in 33 of the 114 (28.95%) the right forelimb was affected, and one horse suffered fractures of both forelimbs. The other cases comprised 14 hindlimb cases (nine right hind, five left hind), two pelvic fractures, two proximal sesamoid bone fractures in which the affected limb was not recorded, one limb in which the third metacarpal bone, fetlock and pastern had sustained fractures but the affected limb was not recorded and one in which no specific diagnosis was given. The forelimb proximal sesamoid bones were fractured in 55.26% of the cases. The second most common CMI location was the diaphysis of the third metacarpus (13.16%) followed by the carpal region at 12.28%. Fractures involving the first phalanx, the third metacarpal condyles and tibia were represented respectively by 6.14%, 4.39% and 2.63% of the cases. Pelvic and scapular fractures each represented 1.75% of the cases. Only one fractured femur occurred (0.88%). In one case, the affected area was not reported (Figure 1).

Of the 53 limbs examined, proximal sesamoid bone fractures represented the most common CMI injury and fracture type (88.7%, 47/53 limbs). Only six cases did not include a proximal sesamoid bone fracture. Most proximal sesamoid bone fractures occurred on the LF (35/47 limbs, 74.47%) and were biaxial (35/47 limbs, 74.47%). The majority of biaxial proximal sesamoid bone fractures occurred in the LF (28/35 cases, 80%). The most common proximal sesamoid bone fracture type was a midbody fracture (39), followed by comminuted (26), basal (13), apical (11), abaxial (two) and axial (one) fractures. The medial proximal sesamoid bone was most commonly affected.

The six remaining limbs had fractures of the third metacarpal condyle, all of which involved the lateral condyle. Of the six third metacarpal condylar fractures, three occurred in the LF and three in the right forelimb. All of them were open fractures. Three of the cases were accompanied by a uniaxial medial proximal sesamoid bone fracture, one by a uniaxial lateral proximal sesamoid bone fracture, with another involving a biaxial proximal sesamoid bone, third metacarpus and first phalanx fractures. One case had no other associated fractures.

Of the 52 horses that sustained a CMI only two of them sustained an open fracture to the diaphysis of the third metacarpus of the LF. One horse suffered a comminuted fracture of the diaphysis of the third metacarpus while the other had multiple fractures including biaxial comminuted proximal sesamoid bone fractures, oblique metaphyseal fracture of the third metacarpus, sagittal diaphyseal fracture of the third metacarpus and an oblique fracture of the lateral condyle.

All the cases with proximal sesamoid bone, third metacarpal or condylar fractures also presented with partial lacerations on the dorsal surface or complete laceration of the suspensory ligament associated with the bone fragments. All the cases showed some degree of deep digital flexor tendon pathology on the dorsal surface. It ranged from mild stretching of fibres and tears to full laceration of the deep digital flexor tendon at the level of the proximal sesamoid bones. It was interesting to note that there did not have to be pathology to the suspensory ligament for there to be visible pathology in the deep digital flexor tendon. Only 14/53 limbs had no pathology to their superficial digital flexor tendons.

## Discussion

The incidence of CMIs in this study falls within the ranges previously reported (Bailey et al. 1998a; Boden et al. 2006; Parkin et al. 2004). The overall incidence of CMIs per 1000 starts for the racing period 1998–2012 was 0.56 (203 965 starters). The four studies that have reported a lower incidence than this included a study in the United Kingdom reporting on the 1999–2001 racing period (0.38), a study in Sydney, Australia, reporting on the 1985–1995 racing period (0.3), a study in Victoria, Australia, reporting on the 1989–2004 racing period (0.44) and a study in New South Wales, Australia, reporting on the 2009–2014 racing period (0.52) (Bailey et al. 1998a; Boden et al. 2006; Parkin et al. 2004; Wylie et al. 2017). There were, however, also ten studies that reported a higher incidence than this study. This incidence of CMI is also lower than that reported previously during the racing period 1987–2008 in South Africa (1.1; Macdonald et al. 2009) but is slightly higher than the incidence reported in the study conducted from 1998 to 2004 (0.53) for the four Gauteng racetracks involved in the study (Cilliers 2009). No correlations were made between the incidence of CMIs and potential causative factors in that study.

In this study CMIs primarily involved the LF, comparing well with previous studies, where the LF was involved in more than 80% of all the CMIs reported (Cilliers 2009; Cohen, Peloso & Mundy 1997; Estberg et al. 1996; Johnson et al. 1994; MacDonald et al. 2009; Peloso, Mundy & Cohen 1994; Peloso et al. 1996; Williams et al. 2001). Forelimbs are more prone to injury because of greater relative forces imposed on them at higher speeds because of the horse’s weight distribution between the forelimbs and hindlimbs of 60:40 (Ross 2003). Considering the pattern of limb placement during different paces, in the canter and gallop there is a moment in which the whole weight of the horse is placed on only one of the forelimbs, possibly predisposing this limb to injury. The addition of the jockey and their position in the saddle during the race may further shift the distribution of weight more onto the forelimbs when in the light seat position during the actual race, potentially increasing the weight-bearing ratio to 70:30 (Ross 2003). Simultaneous front and hind limb injuries were not common (Williams et al. 2001).

A horse will naturally lead with the inside forelimb when rounding a bend to retain balance, resulting in this inside leading limb being loaded more than the outside limb, especially at the four-beat gallop (Peloso et al. 1994). When racing counter-clockwise horses are usually on the left in the turns and on the right lead during the straight (Peloso et al. 1994). Racing and training in Gauteng take place in a clockwise direction, with the horses most likely to lead with the right forelimb in the turn and changing to the left lead on the straight as the right forelimb fatigues. Thus, it would be assumed that the limb most commonly affected would be the right forelimb when racing clockwise and the LF when racing counter-clockwise. However, this and previous studies show that the LF remains the predominantly affected limb (Boden et al. 2006; Cohen et al. 1997; Estberg et al. 1998; Johnson et al. 1994; Macdonald et al. 2009; Peloso et al. 1994), but two of these studies reviewing the causes and factors related to musculoskeletal injuries did not find a specific side prevalence for fractures (Cohen et al. 1997; Johnson et al. 1994). The reason for this phenomenon remains unknown. It may be speculated that for clockwise racing, more strain is placed on the LF after the flying change of leads, where the entire horse’s weight will be borne by the LF. More weight being placed on the medial aspect of the outside limb would not support the LF being injured in a clockwise but not in a counter-clockwise direction. Alternatively, preferential use of a stronger dominant limb by the horse may be related to preferentially sided fatalities. Reviewing racing videos of CMIs occurring may facilitate determining the site on the racetrack where the different limbs are injured and whether a pattern arises for left or right forelimbs. Some locations identified have been associated with a higher frequency of specific injuries (Cohen et al. 1997; Hill, Stover & Gardner 2001; Peloso et al. 1994).

The most common anatomical location for a CMI in this study was the metacarpophalangeal region, including the suspensory apparatus (55.75% of the 114 cases). Unfortunately, the data available did not allow for further classification of these injuries into proximal sesamoid bones or suspensory ligament. The flexor tendons or the suspensory ligament (25.37%), followed by the metacarpophalangeal region and proximal sesamoid bones (11.46%), were reported to be the most common injury sites for CMIs in Britain (Williams et al. 2001). Injuries to the flexor digital tendons other than ante-mortal bruising were not observed in the present study. However, the British study investigated all injuries occurring on racetracks and not specifically CMIs resulting in euthanasia. Most tendon injuries do not result in euthanasia, even if severe enough to end a racehorse’s racing career. In a study conducted in California, the proximal sesamoid bones and the third metacarpus (42% of cases) were the most common racing and training injury sites reported (Estberg et al. 1996). Injuries and damage to the suspensory apparatus were virtually solely seen occurring in the forelimbs of Thoroughbred racehorses that were performing at high speeds (Radtke et al. 2003). The stress experienced by the components of the suspensory apparatus such as the proximal sesamoid bones varies with the racing speed, and the metacarpophalangeal region is at its greatest risk for injury when heavily loaded at full gallop (Radtke et al. 2003).

Fractures of the third metacarpal or third metatarsal condyles occur almost solely in racehorses while the horse is performing at high speeds and predominately involve the lateral condyle of the LF (Parkin et al. 2004; Johnson et al. 1994). These are pathological stress fractures associated with cyclic loading (Radtke et al. 2003). It has been theorised that the high incidence of this fracture results from unbalanced loading on the LF that peaks during counter-clockwise turns. In this study, all six of the condylar fractures involved the lateral condyle and were represented by three LFs and three right forelimbs. Previous studies have shown that condylar fractures predominantly involve the lateral condyle of the LF, 76% lateral versus 8% medial (Johnson et al. 1994) and 0.97 lateral per 1000 starts versus 0.24 medial per 1000 starts in all race types (Parkin et al. 2004). However, the number of limbs available for dissection may not be representative of all CMIs that occurred in the study period. Only the limbs of horses that had obvious pathology to their fetlock were submitted for evaluation, and therefore these did not include all the limbs of horses sustaining a CMI to the distal limb during a race. It is possible that some condylar fractures may have been missed.

### Limitations

The greatest limitation was the small number of horses available for evaluation and dissection. Further, no correlations were made between the incidence rate of CMIs and potential causative factors in this study. As mentioned earlier the classification of fractures was made on site and only via palpation. The use of digital radiography would have greatly enhanced accuracy

## Conclusion

The types of CMIs seen in South Africa are similar to those reported elsewhere, occurring almost exclusively unilaterally and involving mostly the LF. The metacarpophalangeal joint was the predominant CMI location and proximal sesamoid bone fractures the most common fracture. Further studies are required to assess incidence of CMIs at all racetracks in South Africa and to investigate the predominance of LF injuries throughout the world irrespective of racing direction.
